# Intracellular trafficking and endocytosis of CXCR4 in fetal mesenchymal stem/stromal cells

**DOI:** 10.1186/1471-2121-15-15

**Published:** 2014-05-16

**Authors:** Rebecca A Pelekanos, Michael J Ting, Varda S Sardesai, Jennifer M Ryan, Yaw-Chyn Lim, Jerry KY Chan, Nicholas M Fisk

**Affiliations:** 1 UQ Centre for Clinical Research, The University of Queensland, Herston QLD 4029, Australia; 2 Department of Physiology, National University of Singapore, Singapore, Singapore; 3 Department of Reproductive Medicine, KK Women’s and Children’s Hospital, Singapore, Singapore; 4 Experimental Fetal Medicine Group, Department of Obstetrics and Gynaecology, National University of Singapore, Singapore, Singapore; 5 Centre for Advanced Prenatal Care, Royal Brisbane & Women’s Hospital, Brisbane 4029, Australia

**Keywords:** Fetal mesenchymal stromal cells, Bone marrow, MSC, CXCR4, Chemokine receptor, Migration, Small molecule, Endocytosis

## Abstract

**Background:**

Fetal mesenchymal stem/stromal cells (MSC) represent a developmentally-advantageous cell type with translational potential.

To enhance adult MSC migration, studies have focussed on the role of the chemokine receptor CXCR4 and its ligand SDF-1 (CXCL12), but more recent work implicates an intricate system of CXCR4 receptor dimerization, intracellular localization, multiple ligands, splice variants and nuclear accumulation. We investigated the intracellular localization of CXCR4 in fetal bone marrow-derived MSC and role of intracellular trafficking in CXCR4 surface expression and function.

**Results:**

We found that up to 4% of human fetal MSC have detectable surface-localized CXCR4. In the majority of cells, CXCR4 is located not at the cell surface, as would be required for ‘sensing’ migratory cues, but intracellularly. CXCR4 was identified in early endosomes, recycling endosomes, and lysosomes, indicating only a small percentage of CXCR4 travelling to the plasma membrane. Notably CXCR4 was also found in and around the nucleus, as detected with an anti-CXCR4 antibody directed specifically against CXCR4 isoform 2 differing only in N-terminal sequence. After demonstrating that endocytosis of CXCR4 is largely independent of endogenously-produced SDF-1, we next applied the cytoskeletal inhibitors blebbistatin and dynasore to inhibit endocytotic recycling. These increased the number of cells expressing surface CXCR4 by 10 and 5 fold respectively, and enhanced the number of cells migrating to SDF1 in vitro (up to 2.6 fold). These molecules had a transient effect on cell morphology and adhesion, which abated after the removal of the inhibitors, and did not alter functional stem cell properties.

**Conclusions:**

We conclude that constitutive endocytosis is implicated in the regulation of CXCR4 membrane expression, and suggest a novel pharmacological strategy to enhance migration of systemically-transplanted cells.

## Background

Fetal mesenchymal stem/stromal cells (fMSC) have properties intermediate between adult and embryonic stem cells, and thus considerable therapeutic potential. Advantageous characteristics of fetal over adult MSC include their higher proliferative rate, greater differentiation capacity and longer telomeres with reduced senescence [1]. Indeed, a primary fetal bone marrow MSC line from our team had the fastest migratory capacity of 70 cell lines assessed at a recent American Society of Cell Biology (5.2 μm/minute or 0.000000312 kilometers per hour) [2]. Successful translation of cell therapies requires an in-depth understanding not only of the basic stem cell properties of differentiation and self-renewal, but also of the cell type’s adhesive and migratory properties.

MSC can be mobilized from their sedentary niches by a range of external stimuli and triggered to migrate to and occupy distant tissue sites [3]. Preferential homing to sites of tissue injury has been demonstrated with both adult [4,5] and fetal MSC [6-8]. However poor understanding of the underlying processes in adult and particularly fetal MSC limits our ability to exploit them for targeting MSC to regions of tissue damage. Improved homing would facilitate therapeutic development and reduce inappropriate uptake of MSC in healthy tissues.

CXCR4 is a G-protein coupled receptor belonging to the CXC family of chemokine receptors. Interaction of CXCR4 with its ligand, stromal derived factor (SDF-1α, CXCL12) directs the movement of cells in hematopoietic stem cell homing [9], leukocyte trafficking [10,11] and tumour metastasis [12,13]. The CXCR4-SDF-1α axis is active in adult MSC, which migrate toward SDF-1α *in vitro*[14]. CXCR4 is expressed on the surface of only a subset of adult MSC, but up-regulation by viral transduction increases MSC homing [15], rendering CXCR4 a target for modulating migration. However the viral approach is unsuitable for clinical translation due to the risk of insertional mutagenesis. CXCR4 expression is dynamically regulated by external cues like hypoxia [16], and can be up-regulated in adult MSC following *in vitro* priming with a mixture of cytokines, as shown to enhance migration *in vitro* toward an SDF-1α gradient as well as homing *in vivo* to bone marrow [17]. Recently, SDF-1 exposure was shown to up regulate low basal CXCR4 surface expression in fetal blood derived-MSC, which increased chemotaxis [18].

Like other G-protein coupled receptors, CXCR4 undergoes internalization after interaction with ligand. Ligand-induced endocytosis of CXCR4 and its internal sequestration has been extensively studied in leukocytes [19,20] and to a lesser degree in hematopoietic stem cells [21,22] and tumour cells [23]. Although these studies confirm the existence of a general regulatory mechanism, the extent of intracellular expression and endocytosis/recycling kinetics differs between cell types, implicating cellular context in the regulation of CXCR4 trafficking and its functional consequences [24,25]. The predominant intracellular localization of CXCR4 suggests that dynamic equilibrium between the cytoplasm and plasma membrane may modulate CXCR4 availability at the cell surface, and thus fMSC responsiveness to SDF-1α gradients.

We investigated the intracellular localization and trafficking of CXCR4 in fetal bone marrow MSC, and treated fMSC with blebbistatin and dynasore, specific inhibitors of myosin IIA and dynamin subunits of the actin cytoskeleton responsible for cytoskeletal movement and chemotaxis, and commonly associated with G-protein endocytosis. Our findings demonstrate that surface expression of CXCR4 on fMSC and their SDF-1α induced-chemotaxis *in vitro* can be increased through inhibition of receptor endocytosis. These data support further development of small molecule agents to up-regulate the functional expression of a key receptor involved in homing and engraftment of MSC.

## Methods

### MSC culture

Fetal tissue was collected from consenting women undergoing clinically indicated termination of pregnancy in accordance with national guidelines and as approved by the Human Research Ethics Committee of the Royal Brisbane and Women’s Hospital. Early trimester bone marrow MSC (passage 1–7) derived from different donors (n = 9, gestation 10–13 weeks) and adult bone marrow MSC (aMSC) from a bone marrow donor were cultured in Dulbecco’s modified Eagle’s medium (DMEM) high glucose (Invitrogen) supplemented with 10% fetal bovine serum (FBS), 100 IU/mL penicillin, and 100 μg/mL streptomycin (Invitrogen), expanded at 5000 cells/cm^2^ at 37°C with 5% CO_2_. Isolated fMSC and aMSC were characterised by typical cell surface phenotype and differentiation capacity as previously reported [26-28]. Antibodies used to characterize MSC are listed in [28]. Mesodermal differentiation methods are described in the Additional file 1.

### Priming fMSC with endocytosis inhibitors

For flow cytometry, cells were primed with (−)-blebbistatin (1-Phenyl-1,2,3,4-tetrahydro-4-hydroxypyrrolo [2.3-b]-7-methylquinolin-4-one, cat. # B0560, Sigma Aldrich) and dynasore hydrate (3-Hydroxy-naphthalene-2-carboxylic acid (3,4-dihydroxy-benzylidene)-hydrazide hydrate, cat. # D7693, Sigma Aldrich) as follows: cells were detached with TrypLE Select and washed twice with serum-free DMEM. Cells were resuspended in 600 μl of DMEM not containing FCS but with 25 mM HEPES (4 × 10^4^ cells/ml) with blebbistatin or dynasore added at a concentration of 80 μM. Cells were incubated at 37°C for 15, 30 or 60 min. For morphology and immunofluorescence studies, cells were seeded into 24 well plates with or without glass coverslips. When confluent, cells were washed once with DMEM and treated as indicated. Treatment media consisted of 250 μl of DMEM + 25 mM HEPES without FCS and 80 μM blebbistatin or dynasore as necessary. Vehicle treatment was 0.45% DMSO in the same media, equivalent to the amount of DMSO in 80 μM blebbistatin solution. Cells were imaged immediately or washed with PBS, fixed with 1% PFA in PBS pH 7.4 for 10 min, washed and stored in PBS at 4°C.

### Flow cytometry

For flow cytometry, cells were detached using TrypLE reagent (Invitrogen). The antibodies used are detailed in Additional file 1: Table S1. Because of expression differences with different commercially available antibodies, we detected intracellular and extracellular CXCR4 using a monoclonal anti-human CXCR4 PE-Cy5 conjugated antibody (clone 12G5) following the manufacturer’s instructions (1:20 dilution, eBioscience), a non-conjugated polyclonal antibody (clone ab2074, 1:50 dilution, Abcam) and/or a biotin-conjugated monoclonal antibody (clone 4417, 1:20 dilution, R and D Systems). For indirect staining with the polyclonal antibody, cells were incubated with primary antibody at 4°C for 30 min then washed once with PBS + 2% FCS. After washing cells were resuspended and incubated with FITC- or Alexa 488 conjugated secondary anti-rabbit antibody or PE- or FITC labelled streptavidin at 4°C for 30 min. After incubation cells were washed three times with PBS. For intracellular staining for detection of total cellular CXCR4 (surface + cytoplasmic), cells were fixed with 4% paraformaldehyde (Sigma) for 10 min, permeabilised with 0.5% Triton X-100 (Sigma) for 10 min, and stained with anti-human CXCR4-PE Cy5 for 30 min. Cells were analysed by FACS Calibur (Becton Dickinson - fetal MSC, adult MSC and THP-1 cells) or Gallios (Beckman Coulter - fetal MSC and HeLa cells). For flow cytometry experiments the isotype controls, analysis gates were optimized on each flow cytometer, for permeablized or intact cells, and for each antibody, fluorophore and cell line used according to standard practice. For cytoplasmic staining, cells were fixed and permeabilised as above. Cells were then stained with CXCR4-PE-Cy5, and incubated for 5 min in 0.25 M acetic acid (pH 4) to remove surface bound antibody. Surface CXCR4 was expressed as a percentage of total CXCR4 as follows: [MFI (surface CXCR4)/MFI (total CXCR4)] × 100. The CXCR4 antibodies were validated using THP-1 cells (leukemic monocytes, obtained from ATCC), a control cell haematopoietic cell with constitutively high surface expression. Antibodies were also assessed in human cervical carcinoma HeLa cells (ATCC) cultured in MSC medium.

### Immunofluorescence microscopy

fMSC (P3-7) were cultured on glass coverslips and then fixed with 4% PFA in PBS (pH 7.4) for 10 min at room temperature [28]. Cells were washed with PBS, and then incubated with blocking and permeabilisation buffer (2% bovine serum albumin, 0.1% Tween) for 1 hr at room temperature. Blocking buffer was discarded and antibodies were diluted in diluent buffer (blocking buffer diluted 1:10 with PBS) and incubated overnight with primary antibodies at 4°C. Rabbit anti-CXCR4 (ab2074) and either mouse anti-Rab5, anti-Rab11A, or anti-Lamp1 (all 1:50 dilution, detailed in Additional file 1: Table S1) was added. After incubation with primary antibodies, cells were washed 3 × 5 min and secondary anti-mouse-Alexa 568 and anti-rabbit Alexa 488 (1:1000 dilution, from Invitrogen) were added. Cells were incubated with secondary antibodies at room temperature for 1 hr and then washed 3 × 5 min. Coverslips were placed on microscope slides with Prolong Gold with DAPI mounting medium (Invitrogen).

For blebbistatin and dynasore treatment experiments, coverslips were treated with 0.1% gelatin for 10 min prior to seeding or seeded onto fibronectin coated chamber slides. After inhibitor treatment, cells were fixed with prewarmed 1% PFA for 10 min at RT, blocked in 1% BSA with 0.05% Tween in PBS for permeabilisation for 1 hr prior to proceeding with antibody staining. Tween was omitted for surface CXCR4 staining. Antibodies were diluted in PBS. Four x 30 sec washes were carried out after each antibody incubation. Slides were examined on a Zeiss Axio epi-fluorescence or LSM 510 confocal microscope.

### *In situ* Proximity Ligation Assay (PLA)

Cells were cultured on coverslips, fixed with PFA as per Immunofluorescence Microscopy (above). Cells were permeabilised with 0.1% Tween in PBS for 10 min at room temperature. The Duolink II proximity ligation assay (PLA, mouse-rabbit starter kit in orange, Olink Biosciences) was carried out as per manufacturer’s instructions and using supplied reagents for a 1 cm^2^ reaction volume. Primary antibodies were incubated overnight at 4°C - rabbit anti-CXCR4 (1:50, ab2074) and either mouse anti-Rab5 (1:200, 1 μg/ml), anti-Rab11A (1:50, 1 μg/ml) or anti-Lamp1 (1:100, 1 μg/ml) were used. The positive control was the PLA reaction between rabbit anti-Growth Hormone Receptor (GHR) clone H300 (1:200, Santa Cruz) and the mouse anti-GHR clone 3A12 (1:200, Sigma Aldrich), as these 2 different antibodies detect different epitopes of the GHR protein.

### Transwell assays

Fetal MSC were detached using TrypLE reagent (Invitrogen), washed twice in PBS, and resuspended in DMEM. Cells were stained for 8 min at room temperature with CFSE (0.1 mg/ml) and the reaction was then inactivated by adding PBS with 2% FCS. Cells were washed twice and resuspended in DMEM with no FCS. Cells were treated with either endocytosis inhibitors or vehicle only (DMSO) for 1 hr. 300 μl of fMSC suspension (1.6 × 10^4^ cells/well) were placed in the upper compartments of a Fluoroblok transwell migration chamber (BD Biosciences). The transwell membrane (8 μm pore size) was coated with fibronectin 2.5 μg/ml for 30 min at room temperature, then washed with water and allowed to air-dry. SDF-1α was placed in the lower compartment (30, 100 or 200 ng/ml). Cells were allowed to undergo chemotaxis for 4 hours and then migration was determined by fluorescence intensity in the bottom well using the Paradigm fluorescence plate reader (Beckman Coulter). Each experiment was performed in triplicate. The migration index was calculated as the ratio of the fluorescence intensity of cells migrating towards the chemo-attractant to the fluorescence intensity of cells migrating towards media alone.

### Adhesion assays

For adhesion assays, CFSE stained cells (35,000/well) were incubated in fibronectin or collagen I coated wells of 96-well plates in DMEM containing no FBS at 37°C and 5% CO_2_ for 1 hr, and then washed with DMEM without FBS. The number of adherent cells/well was estimated by reading the fluorescence intensity with the Paradigm microplate reader.

### Quantitative wound healing assay

Migration was assessed by performing scratch wound assays in a real-time cell imaging system (IncuCyte Live-cell, ESSEN BioScience Inc.). Briefly, 5 × 10^4^ cells per well were plated into an Essen-Costar 96 well plate coated with 0.1% gelatin (n = 3 independent donors, performed in replicates of 5). Twenty four hours later, the confluent monolayer of cells was washed 1x PBS, scratched with the Essen Bioscience 96 well plate scratcher, incubated with blebbistatin or dynasore as above (80 μM, for 1 hr 37°C). The media was removed and normal growth media added, plates were transferred to the Incucyte live cell imaging system and the Essen 96 well wound assay protocol was run on the software. Cells were imaged at 4 hr intervals for 24 hr to monitor cell migration. Wound confluence was calculated automatically by the Incucyte software (v1.5) for 12 hr, i.e. until wounds were greater than 90% confluent. The data were then analysed using an integrated metric: Relative Wound density.

### Statistical analysis

Normally-distributed data were expressed as mean ± standard deviation and analysed by paired *t*-test and ANOVA. p < 0.05 was considered significant. All experiments were conducted in triplicate or with a minimum of 3 independent donor-derived fMSC, unless otherwise stated. For scratch wound, a 2-way ANOVA with Bonferroni correction was used (Prism, GraphPad).

## Results

### Fetal bone marrow derived-MSC exhibit typical MSC characteristics

Isolated fMSC and aMSC were characterised by typical cell surface phenotype and differentiation capacity as previously reported [26-28]. Cultured MSC met the criteria set by the International Society for Cellular Therapy [29], being plastic adherent, fibroblastic in morphology and able to differentiate osteogenic, adipogenic and chondrogenic lineages *in vitro* (Additional file 2: Figure S1A-D). Fetal MSC were positive for the various MSC markers (including CD73, CD105, CD29, CD90) and were negative for hematopoietic and endothelial markers (including CD45, CD11b, CD34, CD31) (Additional file 2: Figure S1E and F).

### CXCR4 expression is predominantly intracellular and localized to the endosomal, lysosomal and nuclear compartment of fMSC

**Figure 1:**
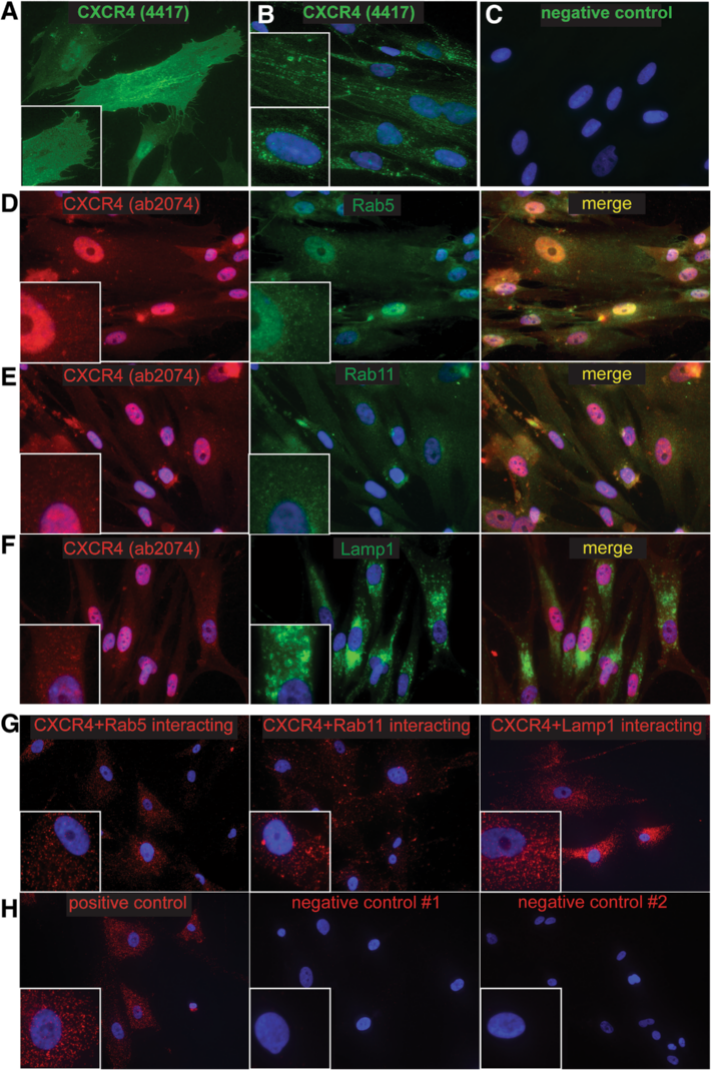
**A low number of human fetal bone marrow MSC (fMSC) display surface CXCR4.** Three different anti-CXCR4 antibody clones were used 12G5, ab2074 and 4417 as detailed in the methods, with fluorophore labelled secondary antibodies used where necessary. **A)** CXCR4 surface expression (fluorescence intensity vs. forward scatter) by flow cytometry shown as dot plots. **B)** After permeabilisation, the majority of cells show intracellular stores of CXCR4. The horizontal gates delineate the position of relevant isotype control antibody. Human adult bone marrow MSC also show low expression of CXCR4 on the cell surface **(C)** with large intracellular stores of this receptor **(D)**. **E)** Each of the anti-CXCR4 antibodies are able to detect >80% cells with surface expression of CXCR4 on HeLa cells. **F)** Both the anti-CXCR4 antibodies 12G5 and ab2074 are able to detect >80% cells with surface expression of CXCR4 on THP-1 human monocytic leukemia cells.

Only 3.8 ± 0.3% of fetal bone marrow-derived mesenchymal stem/stromal cells (fMSC, n = 4) displayed positive surface immune staining for CXCR4 (Figure 1, Additional file 3: Figure S2A). However, when cells were fixed and permeabilised, ~50-90% of fMSC stained positive for intracellularly localised CXCR4 (Figure 1B, Additional file 3: Figure S2B). This low surface and high intracellular CXCR4 staining pattern was observed regardless of the anti-CXCR4 antibody clone used (clone 12G5, 4417 or ab2074) or the method of antibody detection (direct or indirect fluorophore-conjugated primary antibody). Furthermore, detaching fMSC with enzymatic (TrypLE) or chemical methods (5 mM EDTA) produced only a modest change in CXCR4 (12G5) antibody surface staining (3.8% vs 5.3%, Additional file 3: Figure S2H). Therefore, enzymatic degradation of the CXCR4 is not a major reason for low surface CXCR4 expression observed in this study. A similar low surface/high intracellular CXCR4 immuno-positivity was seen for adult bone marrow MSC (passage 5, n = 1, Figure 1C and D), as previously reported [30]. This is in contrast to THP-1 monocytic leukemic and HeLa cell lines, that both displayed a high percentage of cells with surface CXCR4 immuno-positivity (Figure 1E-F, Additional file 3: Figure S2E) and previously reported migration capacity to SDF-1, the CXCR4 ligand [31,32].

**Figure 2:**
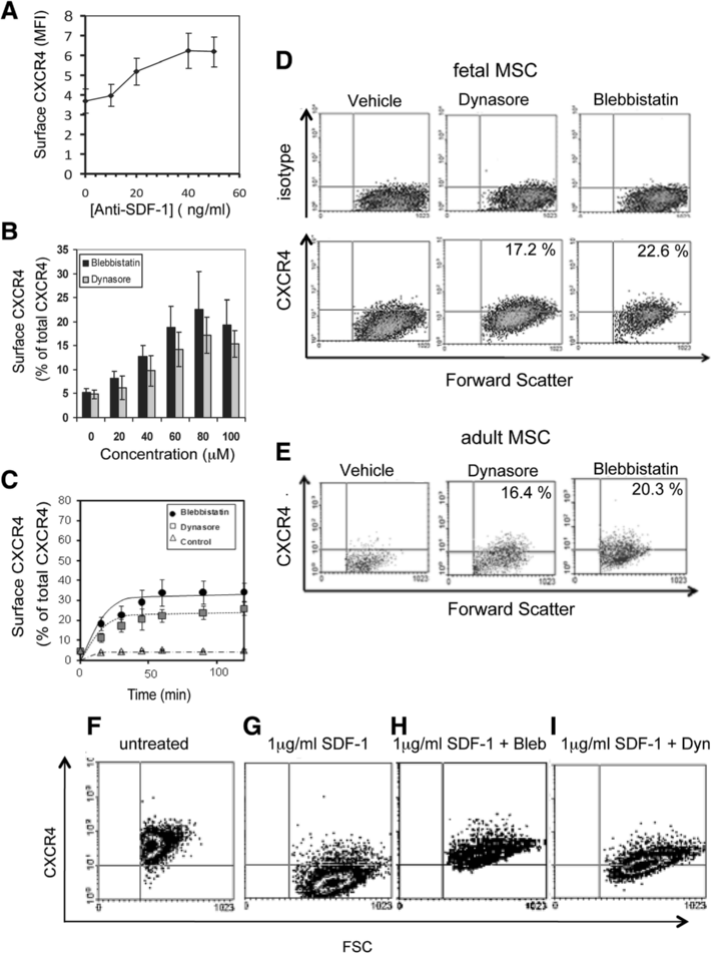
**Subcellular localization of CXCR4 expression in MSC. A)** Incubation of non-permeabilised fMSC with the anti-CXCR4 antibody (clone 4417) shows a distinct plasma membrane labelling of a small percentage of cells (a representative image of positive cell in the centre is shown). **B)** When incubated with permeabilised fMSC, the anti-CXCR4 (4417) antibody labels endosomal-like structures in a majority of cells. These CXCR4 positive vesicles have an arrangement along the cytoskeleton (upper inset) and also perinuclear accumulation (lower inset) with light nuclear staining. **C)** Negative control for ab 4417, using the same imaging settings. **D-F)** Immunofluorescence staining of fMSC with anti-CXCR4 clone ab2074 (red) strong nuclear localization of CXCR4, with diffuse, punctate cytoplasmic staining. CXCR4 colocalises with the endocytotic markers Rab5 **(D)** and Rab11 **(E)** and lysosomal marker Lamp1 (**F**, all green). Lamp1 displays a distinct peri-nuclear location, with larger sized vesicles. Nuclei, counterstained with DAPI (x40 magnification). **G)** The Duolink II proximity ligation assay (PLA) shows colocalisation of CXCR4 (ab2074) with all three Rab5, Rab11 and Lamp1 positive compartments. Each red spot corresponds to a molecular interaction (x20 magnification). **H)** The positive control experiment is two different antibodies to the Growth Hormone Receptor, where the bound antibodies are in close proximity to each other. Negative controls have one (#1) or both (#2) primary antibodies omitted from the PLA procedure.

Although cytoplasmic sequestration of CXCR4 has been described in adult bone marrow and decidual MSC [30], the internal distribution of CXCR4 has not to date been characterised in any MSC. Two CXCR4 antibody clones (4417 and 12G5) detected an even surface distribution of CXCR4 on a small number of fMSC by immuno-fluorescence microscopy (Figure 2A, and not shown). This supported the flow cytometry data that determined only 3.8 ± 0.3% fMSC had positive CXCR4 surface immuno-reactivity (Figure 1). Permeabilization of cells with Triton-X showed CXCR4 to be localized in punctate endosomal/lysosomal-like compartments (Figure 2B), some of which aligned to the cytoskeleton or nuclear membrane (Figure 2B, upper and lower insets respectively). However, the third anti-CXCR4 (ab2074) antibody tested, produced a different pattern of diffuse punctate cytoplasmic staining with a large accumulation of CXCR4 in the nucleus (Figure 2D). Antibody staining conditions were optimized as described in the additional information, but this did not change the nuclear localization of CXCR4 detected by the ab2074 antibody (Additional file 4: Figure S3A-C). We thus examined the localization of CXCR4 using well-characterised markers of the endocytotic pathway, Rab5 and Rab11 for early/recycling endosomes, and Lamp-1 for lysosomes. Immuno-fluorescence staining of fMSC revealed a similar diffuse punctate distribution of anti-CXCR4 (ab2074) antibody with both Rab5 and Rab11 labelled endosomes (Figure 2D and E). Subcellular localization patterns of Rab5 and Rab11 were confirmed by transient transfection of fMSC and HeLa cells (Additional file 4: Figure S3D and E). CXCR4 staining on immuno-fluorescence microscopy also overlapped with Lamp1, although Lamp1 displayed a different peri-nuclear location (Figure 2F).To confirm co-localization of CXCR4 in endosomal compartments, an in cell co-immunoprecipation (proximity ligation assay, PLA) was conducted (Figure 2G). Anti-CXCR4 antibody successfully co-localized with Rab5, Rab11 and Lamp1, with each red spot indicating a point of co-localization. Positive and negative controls for the PLA assay shown in Figure 2H support the specificity of these CXCR4 interactions. These PLA results are consistent with the immunofluorescence microscopy, and indicate that cytoplasmic CXCR4 in fMSC is distributed within all three endosomal compartments.

**Figure 3:**
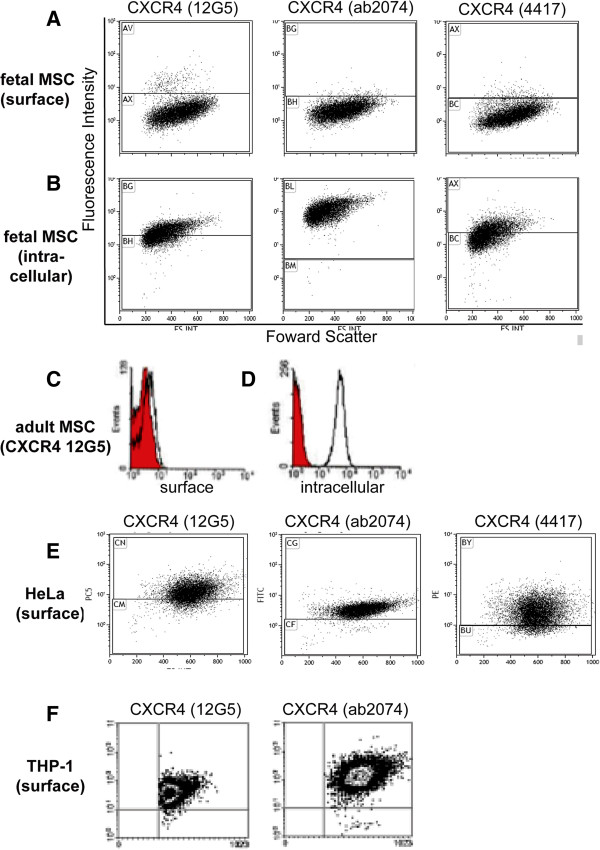
**Surface CXCR4 expression increases after treatment with endocytosis inhibitors. A)** Treatment of fMSC for 24 hr with a neutralizing antibody against SDF-1 at a range of antibody concentrations. fMSC show only low level increase of CXCR4 expression (MFI calculated from flow cytometry data). **B)** Treatment of fMSC with the endocytosis inhibitors, blebbistatin or dynasore increases surface expression of CXCR4. Cells were treated with vehicle (0 μM) or 20–100 μM blebbistatin or dynasore for 60 min before surface expression was determined by flow cytometry (expressed as% total cells expressing surface CXCR4 ± SD). **C)** Kinetics of CXCR4 exocytosis in fMSC after treatment with endocytosis inhibitors. Cells were treated with 80 μM blebbistatin or dynasore then fixed and stained with anti-CXCR4 (12G5) at 0, 15, 30, 60, 90 and 120 min time points. **D)** MSC were incubated with vehicle or 80 μM blebbistatin or dynasore for 60 min. Fetal MSC were stained with isotype control (upper panel) or anti-CXCR4 (12G5, lower panel). **(E)** Inhibitor treated adult bone marrow MSC anti-CXCR4 (ab2074). Percentage of cells positive for CXCR4 expression over isotype control is indicated. Dynasore and Blebbistatin inhibit SDF-1 induced endocytosis of CXCR4 in THP-1 cells. **F)** In the untreated state, anti-CXCR4 antibody 12G5 detected >90% cells with surface expression of CXCR4 on THP-1 monocytic leukemia cells. **G)** Stimulation of THP-1 cells with 1 mg/ml of SDF-1 resulted in down-regulation of surface expression of CXCR4 while co-treatment with either blebbistatin **(H)** or dynasore **(I)** showed reduced CXCR4 endocytosis. The position of the isotype control is indicated by the gates (fluorescence intensity vs. forward scatter).

To investigate what drives internalization of CXCR4, we investigated whether endogenously-produced SDF-1α, the CXCR4 ligand highly expressed in MSC [33], could trigger this endocytosis of CXCR4 through a ligand-dependent process. Fetal MSC expressed very low levels of CXCR4 mRNA, consistent with recycling of receptors rather than synthesis (Additional file 5: Figure S4A). Although fMSC expressed high levels of SDF-1α mRNA (Additional file 5: Figure S4A), treatment of fMSC cultures with a neutralizing antibody against SDF-1α produced only a moderate increase in cell surface expression of CXCR4, as measured by mean fluorescent intensity (MFI) on flow cytometry (Figure 3A). This suggests although ligand-dependent endocytosis occurs, it cannot account for all and particularly basal receptor internalization, and thus an overriding constitutive mechanism is likely to be responsible for the CXCR4 trafficking pattern observed.

### Treatment with endocytosis inhibitors increases CXCR4 surface expression

Given the large proportion of CXCR4 located within the endosomal compartment, we explored the regulatory mechanism responsible by using two inhibitors of endocytosis, blebbistatin and dynasore, to probe the dynamics of CXCR4 trafficking. Blebbistatin is an inhibitor of non-muscle myosin II and inhibits endocytosis, while dynasore is a small molecule inhibitor of dynamin I and dynamin II proteins with important roles in clathrin-dependent endocytosis. fMSC were initially treated with different concentrations (ranging from 20–100 μM) of either blebbistatin or dynasore. After 30 min the highest levels of surface CXCR4 expression determined by flow cytometry (blebbistatin: 22.6% ± 7.9 of cells; dynasore: 17.2% ± 3.8 of cells, n = 3) were achieved at a concentration of 80 μM for both agents (p < 0.01, Figure 3D). Quantitatively similar results were obtained using a different anti-CXCR4 antibody (ab2074) and an indirect staining method on adult MSC (blebbistatin: 20.5% ± 3.0 of cells and dynasore: 16.4% ± 2.4 of cells, Figure 3E). To determine the kinetics of CXCR4 receptor exocytosis, fMSC were treated with 80 μM blebbistatin or dynasore (n = 3 each) at 37°C and the cells then collected at select time points. The cells were stained with CXCR4 antibody (12G5), fixed and the level of surface and cytoplasmic CXCR4 expression examined by flow cytometry. Blebbistatin resulted in a return of CXCR4 to the cell surface with maximal surface expression (33.4% ± 11.3 of cells) reaching steady state after 1 hour (Figure 3C). Dynasore treatment revealed a similar return of surface expression of CXCR4, but with a lower maximal level (25.6% ± 5.4 of cells) compared with blebbistatin. After two hours, CXCR4 surface expression was maintained stably at a level around 5 fold higher than untreated control MSC, but returned to baseline over the subsequent 6 hours (not shown). These endocytosis inhibitors also reduced ligand-induced internalization of CXCR4 in THP-1 cells. This hematopoietic leukemic cell line served as a control cell type with >90% endogenous surface expression when untreated, whereas SDF-1 treatment induced rapid internalization of CXCR4 (Figure 3F and G). However, co-incubation of THP-1 cells with SDF-1 and blebbistatin or dynasore kept the surface levels of CXCR4 similar to untreated (Figure 3H and I).

### Translocation kinetics and modelling indicates CXCR4 endocytosis rate regulates surface expression of CXCR4

At steady state, the relative distribution of a membrane protein between the surface and interior of cells is determined by the ratio of the recycling to endocytosis rate constants. Inhibition of endocytosis in fMSC with blebbistatin and dynasore permits an approximation of a recycling rate (*k*_*r*_) and thus derivation of the endocytosis rate (*k*_*e*_). The high rate of basal endocytosis is consistent with high cytoplasmic sequestration of CXCR4 and the low surface expression we document in fMSC.

Additional file 6: Figure S5B shows the fit of the above model to the experimental data obtained before and after treatment with blebbistatin or dynasore. Simulations using rate constants derived from the experimental data show levels of CXCR4 surface expression of 4.8% for resting fMSC, 35% for blebbistatin and 26% for dynasore. Additional file 6: Figure S5C shows a simulation in which the total cellular CXCR4 is initially present in the intracellular pool. As the system approaches equilibrium, CXCR4 is transferred from the cytoplasmic compartment to the cell surface. Simulations show that the level of membrane expression of CXCR4 in fMSC is sensitive to the rate of endocytosis (*k*_*e*_). The series of theoretical curves generated demonstrate how *k*_*e*_ and *k*_*r*_ inter-determine the level of CXCR4 receptor surface expression and internalization in fMSC. Additional file 6: Figure S5C shows the effect on receptor cell surface expression level of varying *k*_*e*_ while maintaining *k*_*r*_ at its basal rate (0.04 min^−1^). When *k*_*e*_ is small, little internalization is seen and a large proportion of CXCR4 in present on the cell surface (~70%), but as *k*_*e*_ rises, a greater proportion of receptors are found inside the cell at steady state. Thus, fMSC that exhibit small *k*_*r*_ and large *k*_*e*_ have substantial levels of internalization. The model also demonstrates that altering the rate of endocytosis independent of the recycling rate is sufficient to cause substantial receptor re-surfacing. The model supports the rate of endocytosis of CXCR4 being a critical regulatory point for surface expression of CXCR4 in fMSC, and one amenable to manipulation.

### Transient effect of endocytosis inhibitors on fMSC morphology and cytoskeleton arrangement

**Figure 4:**
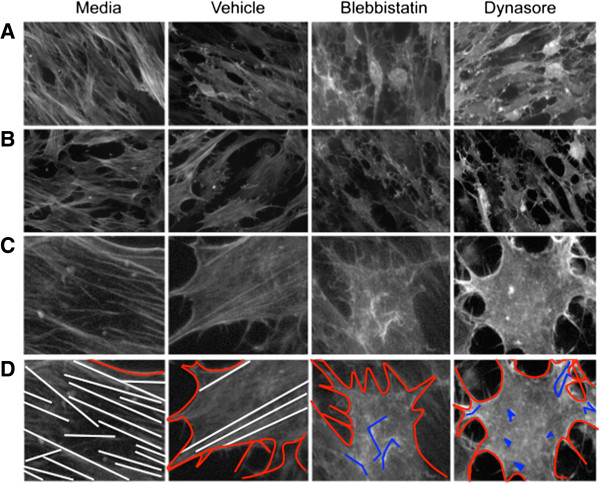
**Endocytosis inhibitors disrupt the actin cytoskeleton of fMSC.** The morphology of the actin cytoskeleton was visualized with Alexa 568-conjugated phalloidin after incubation for 1 hr in serum-free (treatment) media, or treatment media + vehicle, blebbistatin or dynasore. **A)** Cells at high confluence are less prone to cytoskeletal change, whereas cells at lower confluence are impacted more by inhibitors (**B** and **C** shows enlargement of key regions of **B**). **D)** A schematic illustration of **C**, showing actin filaments as white lines, the outline of each cell as a red line, and membrane ruffles as blue lines or spots. A normal, filamentous actin cytoskeleton is seen in fMSC with treatment media. Treatment with vehicle (DMSO) has some impact on cytoskeletal integrity, with the partial loss of long actin filaments in most cells. Blebbistatin and dynasore treatment dramatically disrupts the actin cytoskeleton in most cells. The cells develop membrane ruffles, neural-like projections and begin detaching from the culture dish (x40 magnification).

**Figure 5:**
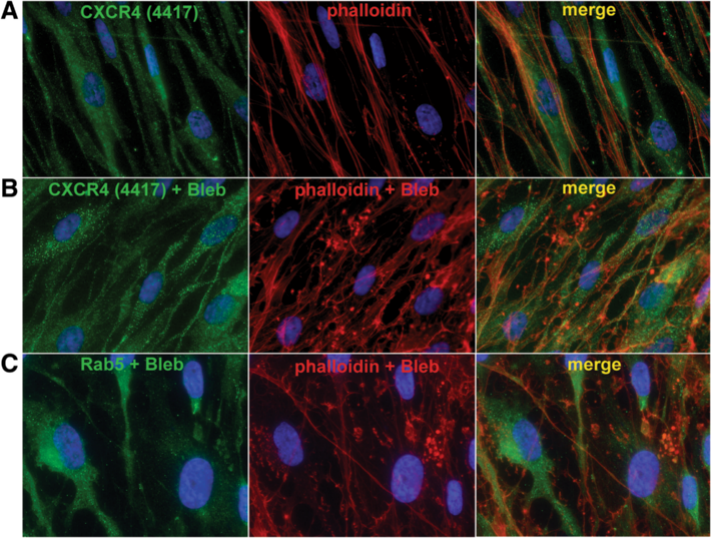
**Blebbistatin does not dramatically alter the morphology of CXCR4 positive endosomes.** The cellular distribution CXCR4 without **(A)** and with blebbistatin treatment **(B). C)** Rab5 distribution with blebbistatin (80 μM, 1 hr). There was little change in the size or distribution of the CXCR4+ or endocytotic vesicles, despite a change in actin cytoskeleton stained with phalloidin (red, **A** vs. **B** and **C**). However, there was a qualitative increase in the amount of staining endosomes trapped throughout the cytoplasm in cells treated with blebbistatin (**A** vs. **B**).

The treatment of blebbistatin and dynasore had a marked effect on the morphology and fragility of cells (Figure 4 and Additional file 6: Figure S5). Inhibitor treatment altered the morphology of the cells’ cytoskeleton within 15 min to a more rounded cell type with projections (Additional file 6: Figure S5), which continued to increase over the 60 min treatment time. However, 24 hr after treatment was stopped, the cells regained their usual shape. This was enhanced by the fact there were no serum or attachment factors in the media and further the vehicle, DMSO, elicited a partial loss of actin cytoskeleton polymerization (Figure 4). The change in structural features in the fMSC with different treatments is illustrated in the schematic shown in Figure 4D. Where a monolayer of cells was less confluent, the inhibitor treatment had a more dramatic effect on morphology than areas with higher cell density (Figure 4A vs. B).When the cellular distribution of CXCR4 and Rab5 following blebbistatin treatment was assessed by microscopy, there was little change in the size or distribution of the endocytotic vesicles, or in the number of CXCR4+ endosomes (Figure 5).

### Partial inhibition of endocytosis does not adversely affect directional migration and can augment CXCR4 function

**Figure 6:**
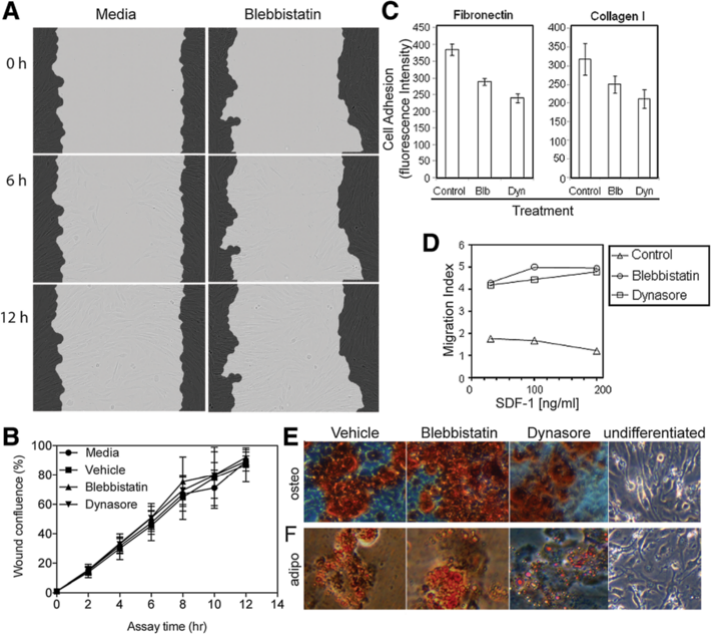
**Effect of endocytosis inhibitors on fMSC migration, attachment, chemotaxis and differentiation. A** and **B)** Scratch wound assay was carried out in a 96 well plate using the Incucyte live cell imaging system. A confluent monolayer of fMSC was wounded and treated for 1 hr with serum-free treatment media, or treatment media with DMSO vehicle, 80 μM blebbistatin or dynasore. Images were taken 4 hourly, with representative images of media and blebbistatin treated cells at 0, 6 and 12 hr intervals shown in **A**. The grey overlay is the automatically generated wound outline at 0 hr. **B)** Quantitative analysis of percentage wound confluence (N = 3 donors, replicates of 5) show no statistically significant difference in ability to migrate into the wound zone for any cell treatment. **C)** fMSC were prestained with CFSE, then treated with either blebbistatin or dynasore before being placed into fibronectin or collagen I coated wells of a 96 well plate. Cell adhesion was determined by fluorescence intensity after a 1 hr incubation and removal of non-adherent cells. **D)** Untreated or inhibitor-treated cells were placed in the upper well of a Transwell plate. Cells were incubated for 4 hr at 37°C and migration determined at a range of concentrations of SDF-1 by fluorescence intensity in the bottom well was higher (p < 0.01** for both) in inhibitor-treated cells. fMSC were treated with either vehicle, 80 μM of blebbistatin or dynasore for 2 hours and the media then replaced with either **(E)** osteogenic- or **(F)** adipogenic-induction media. After 3 weeks culture, differentiation was determined by Alizarin Red (osteogenesis) or Oil Red O (adipogenesis) staining. Undifferentiated fMSC cultured in normal growth media are shown on the right.

We next investigated whether CXCR4 retained functionality after treatment with blebbistatin and dynasore. It had been reported that blebbistatin can disrupt cell migration [34], although other reports suggested it may have little adverse impact on, or even enhance, cellular migration [35,36]. Accordingly we performed a scratch wound assay to determine if pre-treatment of fMSC with either inhibitor alters directional migration. Blebbistatin or dynasore did not affect migration of cells into the *in vitro* wound zone (Figure 6A and B, n = 3 donors).

Adhesion to extracellular matrix (ECM) proteins is important not only for tissue cohesion but also cell migration [37]. Therefore, we assessed adhesion of treated and untreated fMSC to two ECM components, fibronectin and type I collagen. Interestingly, treatment with blebbistatin and dynasore reduced adhesion to both fibronectin and collagen I by up to a third (p < 0.05, n = 3) compared to untreated fMSC (Figure 6C). This suggests a role for these inhibitors in cell mobilisation, likely via disruption of the cytoskeleton (Figure 4). Chemotaxis of fMSC was then examined in a transwell assay. fMSC were either treated with 80 μM blebbistatin or 80 μM dynasore, or control-treated with vehicle (0.45% DMSO). Cells were exposed to the inhibitors for 1 hour to maximize cell surface expression of CXCR4 prior to being placed in the top chamber of Transwell plates. Migration to three different concentrations of SDF-1 (30, 100, and 200 ng/ml) was determined after 4 hours (Figure 6D). Both blebbistatin and dynasore enhanced chemotaxis toward SDF-1α compared to control cells, by over two fold at all concentrations examined (2.6 fold higher than vehicle-treated cells, p < 0.01).

### Effect of endocytosis inhibitors on fMSC viability and differentiation

To exclude any adverse impact of treating fMSC with inhibitors on cell viability and osteogenic/adipogenic differentiation, fMSC were exposed to differing concentrations of blebbistatin for 1 hour, then counted and live/dead discrimination determined. Measures of cell survival including Picogreen assay for DNA content and 7-AAD flow cytometry staining showed no effect of 1 hour of blebbistatin treatment (Additional file 7: Figure S6). Only blebbistatin was tested as this increased the level of surface CXCR4 expression the greatest.

To ensure transient treatment with blebbistatin and dynasore do not have long-term effects on mesenchymal differentiation, fMSC exposed to either blebbistatin or dynasore for 2 hours, and the cells then cultured under standard inductive conditions for 3 weeks. All cultures showed evidence of the relevant osteogenic or adipogenic differentiation after staining with Alizarin Red or Oil Red O (Figure 6E and F). Thus, short-term blebbistatin and dynasore exposure had no lasting detrimental effect on fMSC that would hinder *in vivo* use after inhibitor treatment.

## Discussion

We report a role for CXCR4 receptor trafficking in the migration of first trimester fetal MSC toward SDF-1. fMSC showed a large intracellular pool of CXCR4 protein (Figure 1), but only marginal expression of CXCR4 on the cell surface. In this basal state, internal sequestration of CXCR4 was associated with only modest migration of naïve fMSC toward SDF-1. Immunofluorescence microscopy and *in situ* PLA experiments examining the relationship between CXCR4 and components of the endosomal compartment showed that CXCR4 receptors were present within Rab5+ and Rab11+ endosomes, with another fraction residing within Lamp1+ late endosomes/lysosomes (Figure 2A-C) [38]. The kinetic profile of CXCR4 trafficking in fMSC as supported by translocation modelling indicates that CXCR4 endocytosis rate regulates surface expression of CXCR4. Together this indicates a state of accelerated internalization of CXCR4 in resting fMSC (Figure 3), which when relieved by inhibition of endocytosis led to an increase in CXCR4 surface expression and potentiated fMSC migration toward an SDF-1α gradient in Transwell assays (Figure 6D).

Flow cytometry data indicate that there was up to 10-fold increased CXCR4 expression at the cell surface after blebbistatin treatment (Figure 3B and D). However this was difficult to confirm conclusively with microscopy due to the differences in sensitivity between the two techniques and the low total expression of CXCR4 in fMSC, or alternatively perhaps because inhibiting endocytosis is not the main mechanism of the increase in surface CXCR4. This 5-10-fold increase in surface CXCR4 expression, with a 2.6 fold increase in migration index, is superior to what has been reported for genetic over-expression of CXCR4. Recently Marquez-Curtis *et al*. reported that non-transfected human fetal umbilical cord blood-derived (UCB)-MSC had <2% CXCR4+ population [9]. However, after transiently transfecting with a CXCR4 expression construct, 40% of cells were CXCR4+, resulting in a ~3-fold increased migration to SDF-1. Furthermore, Marquez-Curtis *et al*. claim their transient transfection method in fetal UCB-MSC produced superior results to other expression methods in adult rat MSC, which although increased CXCR4 expression to 54-95% of cells, but showed a mere 2–3 fold increased migration to SDF-1 [9,15,39].

The marked effect of blebbistatin and dynasore on cytoskeletal morphology is not unexpected as mechanistically they transiently disrupt cytoskeletal components myosin IIA and dynamin [40]. Therefore, a small molecule with reversible effects, such as blebbistatin, is likely to be more effective at enhancing migration rather than over expression or knock down approaches to a single chemokine receptor/ligand. However, this cytoskeletal disruption also makes the cells less adherent and more sensitive to handling.

Although we only used one adult MSC donor in this study, the small number of cells expressing CXCR4 at the plasma membrane was similar to what we found with nine fetal MSC donors and as reported by others for adult MSC [30]. Surface expression of CXCR4 reported in adult MSC has been variable, in the range of 2-25% of cells [41-43]. In keeping with this, there was a disparity between the 4% of cells we found with surface CXCR4 expression in first trimester bone marrow fMSC compared to the Jones *et al*. figure of 23% in blood-derived fMSC [18]. Indeed discrepancies on levels of other fMSC markers in particular Oct4, between our collaborating laboratories was the subject of a recent review [44]. This difference in CXCR4 expression might reflect inherent biological variation in MSC samples (donor or organ sourced, age, sampling method) coupled with differing methods of culture (serum batch) or different antibodies used in analysis.

Our finding of nuclear localized CXCR4 in bone marrow fMSC is consistent with recent findings in blood fMSC [18]. The human CXCR4 contains a nuclear localization motif [45] and nuclear CXCR4 translocation has been reported to be a negative prognostic marker in several highly proliferative cancers [46-49]. Furthermore, a number of studies have found, similar to our study, that different antibody clones against CXCR4 can show disparate subcellular localization patterns, including some with nuclear localization and others with cytoplasmic localization of CXCR4 [50]. This could be due to the epitope recognised by the antibody; for example the epitope of the CXCR4 ab2074 antibody, which we found to detect the nuclear CXCR4, is the N-terminal 20 amino acids, 9 residues of which differ between the CXCR4 mRNA splice variants 1 and 2 [51,52]. A number of molecular weight forms of CXCR4 protein have been described by western blotting of various cells and tissues, which have been determined to be due to splice variants, dimeric receptors, and post-translational modifications [32,53].

Fetal MSC express both CXCR4 and its ligand SDF-1 as confirmed here. Reports of an inverse correlation between CXCR4 and SDF-1 expression by MSC [54], is one possible explanation for our finding. That is, endogenously produced SDF-1 binds surface CXCR4, induces internalization of CXCR4, and potentially forms a suppressive autocrine loop down regulating CXCR4 expression. To investigate this we showed that treatment with a neutralizing antibody against SDF-1, resulted in only a small increase in surface expression of CXCR4 (p < 0.01). Furthermore, prolonged treatment or increased antibody concentration, which would be expected to lessen autocrine suppression, did not restore CXCR4 surface expression to a substantial level. Thus while autocrine SDF-1 may trigger some ligand-dependent CXCR4 internalization, this is not the principal mechanism responsible for intracellular localization of CXCR4 in fMSC. These data also support previous findings in non-MSC cell lines [53].

The chemokine receptor and other migratory mechanisms in MSC are not well understood, leading to seemingly contradictory findings in the literature [55-61]. This may be due to different migratory assays detecting different type of cellular movements. Similarly, we found that blebbistatin and dynasore did not have any effect on two dimensional migration in the scratch wound assay, but did have a significant increase in the number of cells that migrated in transwell assay in response to SDF-1. In the transwell assay, cells migrate through a membrane in response to a chemotactic ligand (e.g. SDF-1), mimicking an *in vivo* injury paradigm. Different migratory effects observed may also depend on the suite of chemokine receptors and ligand isoforms expressed by MSC, and the *in vitro* or *in vivo* environment [58]. Furthermore, a number of studies ignore the capacity of CXCR4 to cross talk with other receptors directly or indirectly, especially the heterodimerizing CXCR4-CXCR7 pair [41,62] or have alternative ligands [57,63]. Nor do they take into account that the ligands of CXCR4 and CXCR7 homo- and heterodimeric complexes, SDF-1 and MIF, are highly expressed by MSC [64]. Park *et al.* demonstrated CXCR4-overexpressing MSC displayed enhanced migration to SDF-1, but more so to glioma-conditioned media, which contains a multitude of migratory factors [65].

## Conclusions

In conclusion, this study is the first to propose a reversible, small molecule method for enhancing MSC migration. Understanding the novel role of the cytoplasmic and nuclear localised CXCR4 described here may further augment fetal MSC migration for translation therapeutic uses.

## Abbreviations

CXCR4: chemokine receptor C-X-C motif 4; F: fetal; MSC: mesenchymal stem or stromal cell; SDF-1: stromal derived factor-1; PLA: Proximity Ligation Assay.

## Competing interests

The authors report no potential conflicts of interests.

## Authors’ contributions

MT was responsible for the original concept, carried out experimental design, data acquisition and analysis, especially in relation to the blebbistatin and dynasore treatment experiments and mathematical modelling. RP carried out experimental design, data acquisition, analysis, and interpretation for morphology, immunofluorescence, PLA, wound assay, viability and mesodermal characterization. RP and VS carried out flow cytometry, viability and morphology experiments. VS and JR collected samples, and helped characterize MSC. LYC and JC undertook parallel investigations in Singapore and assisted with data analysis and interpretation. NF assisted MT in study design and data analysis and coordinated the research. MT, RP, JC and NF wrote the paper. All authors have read and approve the final manuscript.

## Supplementary Material

Additional file 1 Pelekanos et al. Supplementary Text R1. Click here for file

Additional file 2: Figure S1 Characterization of fetal Mesenchymal Stem/Stromal Cells. **A)** Light microscopy (x10 magnification) of fetal MSC showing an adherent, fibroblast like morphology. Fetal MSC display osteogenic **(B)**, adipogenic **(C)** and chondrogenic **(D)** differentiation capacity after incubation with specific induction media and staining with Alizarin Red, Oil red-O or Alcian Blue respectively (x4, x10 and *x*2 magnification respectively). **E)** Flow cytometry for MSC positive markers: CD73, CD105, CD90, CD44, HLA-ABC, CD29, CD49b, CD49d. **F)** Flow cytometry for MSC negative markers: CD11b, CD34, CD45, CD117, CD31, HLA-DR, CD14. Fluorophore conjugates are indicated. Click here for file

Additional file 3: Figure S2 Flow cytometry of CXCR4 expression by MSC control cell lines. Three different anti-CXCR4 antibody clones were used 12G5, ab2074 and 4417 as detailed in the methods, with fluorophore labelled secondary antibodies used where necessary. **A)** Fetal MSC CXCR4 surface expression (cell count vs fluorescence intensity) by flow cytometry shown as histograms plots (same data as for Figure 3). **B)** After permeabilization, the majority of cells show intracellular stores of CXCR4. Red histogram indicates isotype control. Human adult bone marrow MSC also show low expression of CXCR4 on the cell surface **(C)** but large intracellular store of this receptor **(D)**. **E** and **F)** All the anti-CXCR4 antibodies are able to detect >80% cells with surface expression of CXCR4 on HeLa human cervical cancer cells. **G)** Both the anti-CXCR4 antibodies 12G5 and ab2074 are able to detect >80% cells with surface expression of CXCR4 on THP-1 human monocytic leukaemia cells. **H)** Fetal MSC were detached with TrypLE trypsin replacement or 5 mM EDTA and stained with CXCR4 (12G5) antibody to assess the effect of enzymatic dissociation on the number of cells staining positive for surface CXCR4. Click here for file

Additional file 4: Figure S3 Optimization of the fixation and permeabilisation conditions for the anti-CXCR4 (ab2074) antibody in fMSC or HeLa cells. **(A** and **B)** fMSC or HeLa cells were fixed and permeabilised as follows (from left to right): methanol, 4% paraformaldehyde (PFA) + 0.3% Triton and PFA + 0.1% Tween, and then stained with CXCR4 (ab2074) as detailed in the methods section. The negative control was PFA + Triton treated cells but the primary CXCR4 antibody omitted. **C)** HeLa cells with the anti CXCR4 antibody clone ab2074 or 12G5, where the cells were fixed in PFA to show surface staining or treated with PFA + Triton to show intracellular staining. **D** and **E)** Fetal MSC and HeLa cells were transiently transfected with Rab5-GFP or Rab11-GFP constructs to ensure the accuracy of the Rab antibody staining. Note that Rab5-GFP shows classic punctate endosomal localization in both fMSC and HeLa, whereas the Rab11 shows diffuse cytoplasmic localization in fMSC and classical endosomal punctae in the HeLa cells, similar to antibody staining in Figure 2. Click here for file

Additional file 5: Figure S4 Two compartment modelling of CXCR4 trafficking. **(A)** Real time PCR for SDF-1: Expression of SDF-1 in fMSC (n = 4) shows SDF-1 and CXCR4 transcripts relative to the housekeeping gene GAPDH. **B)** Kinetics of CXCR4 exocytosis in fMSC after treatment with endocytosis inhibitors: Cells were treated with 80 μM blebbistatin or 80 μM dynasore then fixed and stained with anti-CXCR4. Surface expression was determined by flow cytometry and data fitted to a two-compartment model of endocytosis (same data as Figure 1C). **C)** Fit of CXCR4 surface expression data for naïve (*k*_*r*_ = 0.04 min^−1^, *k*_*e*_ = 0.79 min^−1^), blebbistatin- (*k*_*r*_ = 0.04 min^−1^, *k*_*e*_ = 0.078 min^−1^, r^2^ = 0.89) and dynasore-treated cells (*k*_*r*_ = 0.03 min^−1^, *k*_*e*_ = 0.09 min^−1^, r^2^ = 0.91) to the two compartment model. Simulation was initiated with cytoplasm (endosomes) containing the entire cellular CXCR4. Response of surface expression to changes in the endocytosis rate (*k*_*e*_): *k*_*r*_ was maintained at 0.04 min^−1^ while *k*_*e*_ was varied from 0.79 min^−1^ to 0.02 min^−1^. **D)** Data that was used in the mathematical modelling of CXCR4 trafficking. Click here for file

Additional file 6: Figure S5 Endocytosis inhibitors have a rapid but transient effect on fMSC morphology. Light microscopy images show morphology of fMSC rapidly changes from wide flattened fibroblast-like state (far left panels, 0 min) to cells that become rounded in the centre with long projections after vehicle (DMSO), blebbistatin and dynasore treatment. Cell morphology returns to normal by 24 hr post treatment and removal of the reagents (x10 magnification). Click here for file

Additional file 7: Figure S6 Comparison of fMSC survival after inhibitor treatment. **A)** The Picogreen assay showed that there was no significant difference in the number of cells in control (serum free media), vehicle (0.45% DMSO) conditions or with increasing dose of blebbistatin for 1 hr. **B)** 7-AAD dye exclusion analysed by flow cytometry showed no significant difference between control media (vehicle) and blebbistatin treated fMSC after 1 hr incubation. Click here for file
