# Supplementary material for: Intracellular trafficking and endocytosis of CXCR4 in fetal mesenchymal stem/stromal cells
Source: BMC Cell Biol. 2014 May 16;15:15. doi: 10.1186/1471-2121-15-15 (PMC4065074; doi:10.1186/1471-2121-15-15)
Supplement: Additional file 1 — Pelekanos et al. Supplementary Text R1. [file 1471-2121-15-15-S1.docx]

**Pelekanos et al. Supplementary Text**

**Supplementary Methods and Materials**

**Differentiation assays**

*Osteogenic differentiation*

MSC seeded in 24 well plates were cultured in DMEM, 10% FCS and 2 mM glutamine until 90% confluence. Cells were treated with one of (i) vehicle (DMSO) alone (ii) 80 µM dynasore or (iii) 80 µM blebbistatin for 2 hours at 37°C in serum free DMEM prior to addition of differentiation media. Osteogenic differentiation was induced by culturing cells for 21 days in induction media containing dexamethasone, β-glycerophosphate, L-ascorbic acid-2-phosphate and DMEM-HG (5 g/L glucose). For a positive control, PBS-treated cells were maintained in osteogenic induction medium. The media in all groups were changed every 4 days. Osteogenesis was evaluated by Alizarin Red staining. Following aspiration of media, cells were washed once with PBS, and fixed in PBS with 4 % paraformaldehyde for 30 min at room temperature. Following fixation cells were rinsed with distilled water for a few minutes, and incubated with isopropanol containing an Alizarin Red aqueous working solution. The wells were viewed using an inverted phase contrast microscope.

*Adipogenic differentiation*

MSC seeded in 4 wells of a 24 well plate were cultured in cell expansion media until 100% confluence. Cells were similarly treated with vehicle alone, 80 µM dynasore or 80 µM blebbistatin for 2 hours at 37°C prior to addition of differentiation media. Adipogenesis was induced by culturing cells for 23 days in induction media containing h-Insulin (recombinant), L-Glutamine, dexamethasone, indomethacin, IBMX (3-isobuty-l-methyl-xanthine), 100 IU/mL penicillin, and 100 µg/mL streptomycin. As a positive control, PBS-treated cells were maintained in adipogenic induction medium. Media in all groups were changed every 3 days. Adipogenesis was evaluated by Oil Red O staining (30 mL of 0.5% isopropanol/ Oil Red O solution with 20 mL of distilled water).

**Quantitative Real-Time PCR**

Total RNA (1-2 μg) was treated with RNase-free RQ1 DNase I (Promega Corp., Australia) for 30 minutes at 37°C to remove any possible contamination by genomic DNA. The reaction was terminated by adding 1 μl of DNase I stop buffer (10 mM EGTA, Promega Corp, Australia) and heating the sample for 5 minutes at 65°C. For quality control a small aliquot (1 μl) of the DNase digested sample was reserved for use as a template in a PCR to confirm the absence of DNA. First strand cDNA was prepared by adding 1.5 μl of 500 μg/ml Oligo-dT30, 1.5 μl of 10 mM dNTPs, and 7 μl of DEPC-water to 10 μl of the DNase-digested RNA sample. The mixture was incubated at 65°C. After 5 minutes 6 μl of reverse transcriptase buffer (195 mM Tris-HCl pH 8.3, 37 mM KCl), 1.5 μl of dithiothreitol (DTT), 1 μl of RNasin (40 U/μl, Promega) and 1.5 μl Superscript III reverse transcriptase (Invitrogen) were added and the samples incubated at 50°C for 2 hours. Reactions were stopped by heating the mixture for 15 minutes at 70°C. Quantitative PCR (QPCR) was performed using Quantitect™ SYBR Green PCR master mix (Qiagen, Australia). Each sample was assayed in duplicate on a Corbett Research Rotor-Gene 6000 (Corbett Research, Australia). Reaction parameters comprised a 10 minute 95ºC activation step, 45 cycles of 30 seconds at 95ºC, 30 seconds at 55ºC and 30 seconds at 72ºC with fluorescence recorded at the end of each 72ºC step using the FAM/Sybr Green I channel. Primers were CXCR4 Forward: 5’ gaagctgttggctgaaaagg 3’ Reverse: 5’ ctcactgacgttggcaaaga 3’, SDF-1α Forward: 5’ gggctccctgtaacctcttc 3’ Reverse: 5’ acacacagccagtcaacgag 3’, GAPDH Forward: 5’ acagtcagccgcatcttctt 3’ Reverse 5’ acgaccaaatccgttgactc 3’.

**Computational modelling of CXCR4 trafficking**

A mathematical model was used to examine the distribution of CXCR4 between the plasma membrane and the intracellular pool. The cell membrane and cytoplasmic CXCR4 are in dynamic equilibrium. The proportion of the total CXCR4 receptor pool present on the cell surface can be described by the following linear differential equation.

Where C_m_ is the CXCR4 on the cell surface (plasma membrane), C_tot_ is the total cellular pool of CXCR4, and k_r_ and k_e_ are the recycling rate and endocytotic rate, respectively. The total number of receptors is assumed to be constant (rate of de novo synthesis is equal to receptor degradation) and so C_m_ + C_cyto_ = C_tot_=1, where C_cyto_ is the CXCR4 receptors present within the cytoplasm. Equation 1 can be solved analytically to give equation 2, the time dependent change of CXCR4 at the cell membrane.

In equation 2, as $t\to\infty$ the exponential term reduces to zero and the steady state of surface CXCR4 is represented by equation 3.

The recycling rate constant for CXCR4 in fMSC was estimated by fitting recycling curves generated after treating fMSC with blebbistatin or dynasore (Sup. Fig. 4D). The endocytosis rate was derived from the equilibrium distribution of CXCR4 (steady state surface expression) in fMSC determined from flow cytometry data and the estimated recycling rate constant. Simulations were run using the dynamical analysis software XPPAUT. Parameter estimation and curve fitting was performed using non-linear regression in the statistical software R (<http://www.r-project.org/).>

**Cell Viability and Picogreen assay**

The viability of fMSC after blebbistatin and dynasore or vehicle treatment was determined with 7-AAD exclusion staining. The Picogreen assay (Invitrogen) was used as another measurement of cell survival as per manufacturer’s instructions. Briefly, 2x10^5^ in a Nunc 96 well plate (n=4 independent donors, in quadruplicate) in 20 μM, 60 μM or 80 μM blebbistatin as above for 1 hr at 37°C. Wells were washed with PBS and stored at -20°C. Then the Picogreen reagent was added and fluorescence was measured on the Paradigm microplate reader as per the manufacturer’s instructions.

**Transient transfection**

Fetal MSC and HeLa Cells were transfected with Lipofectamine LTX reagent and OptiMEM media as per manufacturers instructions. Cells were plated so they were approx. 80% confluent at time of transfection. Cells were transfected with 3μl Lipofectamine and 1μl Plus reagent and 500 ng plasmid DNA per well in a 24 well plate (+ 0.1% gelatin coated coverslips). Samples were fixed in 4% PFA 24 hr after transfection. Rab5-GFP and Rab11-GFP plasmids were kindly provided by Prof. Jennifer Stow (Institute for Molecular Bioscience, The University of Queensland, Australia).

**Supplementary Figure Legends**

**Figure S1. Characterization of fetal Mesenchymal Stem/Stromal Cells.**

**A)** Light microscopy (x10 magnification) of fetal MSC showing an adherent, fibroblast like morphology. Fetal MSC display osteogenic **(B)**, adipogenic **(C)** and chondrogenic **(D)** differentiation capacity after incubation with specific induction media and staining with Alizarin Red, Oil red-O or Alcian Blue respectively (x4, x10 and x2 magnification respectively). **E)** Flow cytometry for MSC positive markers: CD73, CD105, CD90, CD44, HLA-ABC, CD29, CD49b, CD49d. **F)** Flow cytometry for MSC negative markers: CD11b, CD34, CD45, CD117, CD31, HLA-DR, CD14. Fluorophore conjugates are indicated.

**Figure S2. Flow cytometry of CXCR4 expression by MSC control cell lines.**

Three different anti-CXCR4 antibody clones were used 12G5, ab2074 and 4417 as detailed in the methods, with fluorophore labelled secondary antibodies used where necessary. **A)** Fetal MSC CXCR4 surface expression (cell count vs fluorescence intensity) by flow cytometry shown as histograms plots (same data as for Fig. 1). **B)** After permeabilization, the majority of cells show intracellular stores of CXCR4. Red histogram indicates isotype control. Human adult bone marrow MSC also show low expression of CXCR4 on the cell surface **(C)** but large intracellular store of this receptor **(D)**. **E and F)** All **t**he anti-CXCR4 antibodies are able to detect >80% cells with surface expression of CXCR4 on HeLa human cervical cancer cells. **G)** Both the anti-CXCR4 antibodies 12G5 and ab2074 are able to detect >80% cells with surface expression of CXCR4 on THP-1 human monocytic leukaemia cells. **H)** Fetal MSC were detached with TrypLE trypsin replacement or 5mM EDTA and stained with CXCR4 (12G5) antibody to assess the effect of enzymatic dissociation on the number of cells staining positive for surface CXCR4.

**Figure S3. Optimization of the fixation and permeabilisation conditions for the anti-CXCR4 (ab2074) antibody in fMSC or HeLa cells.**

**A and B)** fMSC or HeLa cells were fixed and permeabilised as follows (from left to right): methanol, 4% paraformaldehyde (PFA)+0.3% Triton and PFA+0.1% Tween, and then stained with CXCR4 (ab2074) as detailed in the methods section. The negative control was PFA+Triton treated cells but the primary CXCR4 antibody omitted. **C)** HeLa cells with the anti CXCR4 antibody clone ab2074 or 12G5, where the cells were fixed in PFA to show surface staining or treated with PFA+Triton to show intracellular staining. **D and E)** Fetal MSC and HeLa cells were transiently transfected with Rab5-GFP or Rab11-GFP constructs to ensure the accuracy of the Rab antibody staining. Note that Rab5-GFP shows classic punctate endosomal localization in both fMSC and HeLa, whereas the Rab11 shows diffuse cytoplasmic localization in fMSC and classical endosomal punctae in the HeLa cells, similar to antibody staining in Fig. 2.

**Figure S4. Two compartment modelling of CXCR4 trafficking.**

**A)** Real time PCR for SDF-1: Expression of SDF-1 in fMSC (n=4) shows SDF-1 and CXCR4 transcripts relative to the housekeeping gene GAPDH. **B)** Kinetics of CXCR4 exocytosis in fMSC after treatment with endocytosis inhibitors: Cells were treated with 80 μM blebbistatin or 80 μM dynasore then fixed and stained with anti-CXCR4. Surface expression was determined by flow cytometry and data fitted to a two-compartment model of endocytosis (same data as Fig. 3C). **C)** Fit of CXCR4 surface expression data for naïve (*k_r_* =0.04 min^-1^, *k_e_* =0.79 min^-1^), blebbistatin- (*k_r_*=0.04 min^-1^, *k_e_*=0.078 min^-1^, r^2^=0.89) and dynasore-treated cells (*k_r_*=0.03 min^-1^, *k_e_* =0.09 min^-1^, r^2^=0.91) to the two compartment model. Simulation was initiated with cytoplasm (endosomes) containing the entire cellular CXCR4. Response of surface expression to changes in the endocytosis rate (*k_e_*): *k_r_* was maintained at 0.04 min^-1^ while *k_e_* was varied from 0.79 min^-1^ to 0.02 min^-1^**. D)** Data that was used in the mathematical modelling of CXCR4 trafficking.

**Figure S5. Endocytosis inhibitors have a rapid but transient effect on fMSC morphology.**

Light microscopy images show morphology of fMSC rapidly changes from wide flattened fibroblast-like state (far left panels, 0 min) to cells that become rounded in the centre with long projections after vehicle (DMSO), blebbistatin and dynasore treatment. Cell morphology returns to normal by 24 hr post treatment and removal of the reagents (x10 magnification).

**Figure S6. Comparison of fMSC survival after inhibitor treatment.**

**A)** The Picogreen assay showed that there was no significant difference in the number of cells in control (serum free media), vehicle (0.45 % DMSO) conditions or with increasing dose of blebbistatin for 1 hr. **B)** 7-AAD dye exclusion analysed by flow cytometry showed no significant difference between control media (vehicle) and blebbistatin treated fMSC after 1 hr incubation.

Table S1. Antibodies used for flow cytometry and immuno-fluorescence.

| **Antibody** | **Fluorochrome** | **Supplier** | **Catalogue #** |
| --- | --- | --- | --- |
| CXCR4 (clone 12G5) | PE-CY5 | eBiosciences | 15-9999 |
| CXCR4 (clone 4417) | biotin | R&D Systems | FAB173B |
| CXCR4 (ab2074) | none | Abcam | Ab2047 |
| CXCR7 | PE | R&D Systems | FAB42271P |
| Rab5 (clone Rab5-65) | none | Abcam | Ab50523 |
| Rab11A (clone 47) | none | Abcam | Ab78337 |
| Lamp1 (clone H4A3) | none | Abcam | Ab25630 |
| GHR (clone H300) | none | Santa Cruz | sc-20747 |
| GHR (clone 3A12) | none | Sigma Aldrich | WH0002690M1 |
| Anti-mouse IgG (secondary antibody) | Alexa 488 or 568 | Invitrogen | A11092, A11031 |
| Anti-rabbit IgG (secondary antibody) | Alexa 488 or 568 | Invitrogen | A11036, A11089 |
| streptavidin | FITC | BD Pharmingen | 554060 |
| streptavidin | PE | eBioscience | 12-4317-87 |
| phalloidin | Alexa 568 | Invitrogen | A22283 |
