# Supplementary figures and images for: Intracellular trafficking and endocytosis of CXCR4 in fetal mesenchymal stem/stromal cells
Source: BMC Cell Biol. 2014 May 16;15:15. doi: 10.1186/1471-2121-15-15 (PMC4065074; doi:10.1186/1471-2121-15-15)

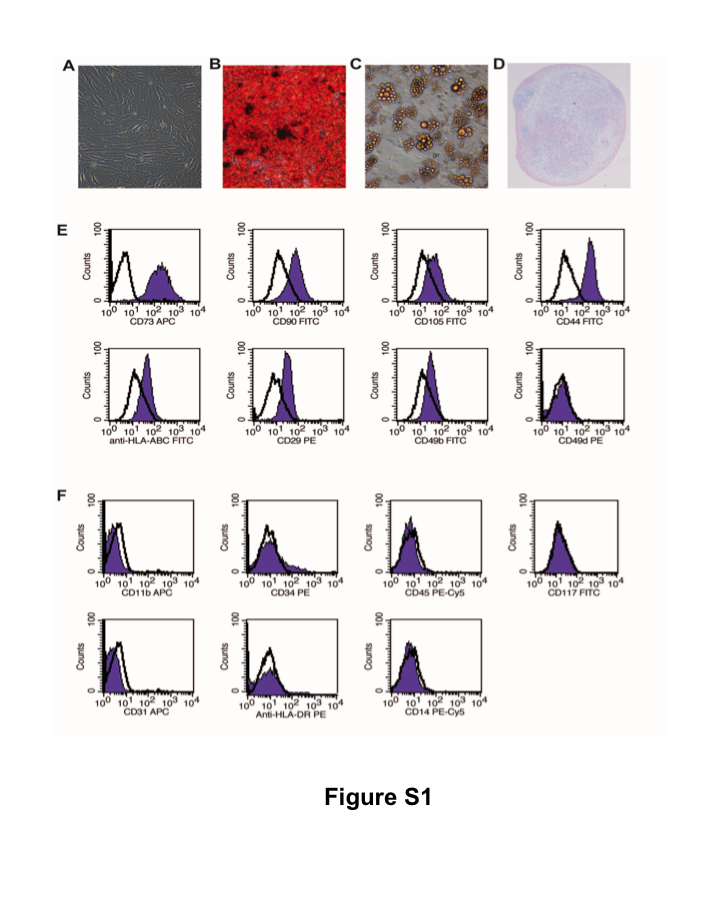

Supplement: Additional file 2: Figure S1 — Characterization of fetal Mesenchymal Stem/Stromal Cells. A) Light microscopy (x10 magnification) of fetal MSC showing an adherent, fibroblast like morphology. Fetal MSC display osteogenic (B), adipogenic (C) and chondrogenic (D) differentiation capacity after incubation with specific induction media and staining with Alizarin Red, Oil red-O or Alcian Blue respectively (x4, x10 and x2 magnification respectively). E) Flow cytometry for MSC positive markers: CD73, CD105, CD90, CD44, HLA-ABC, CD29, CD49b, CD49d. F) Flow cytometry for MSC negative markers: CD11b, CD34, CD45, CD117, CD31, HLA-DR, CD14. Fluorophore conjugates are indicated. [file 1471-2121-15-15-S2.tiff]

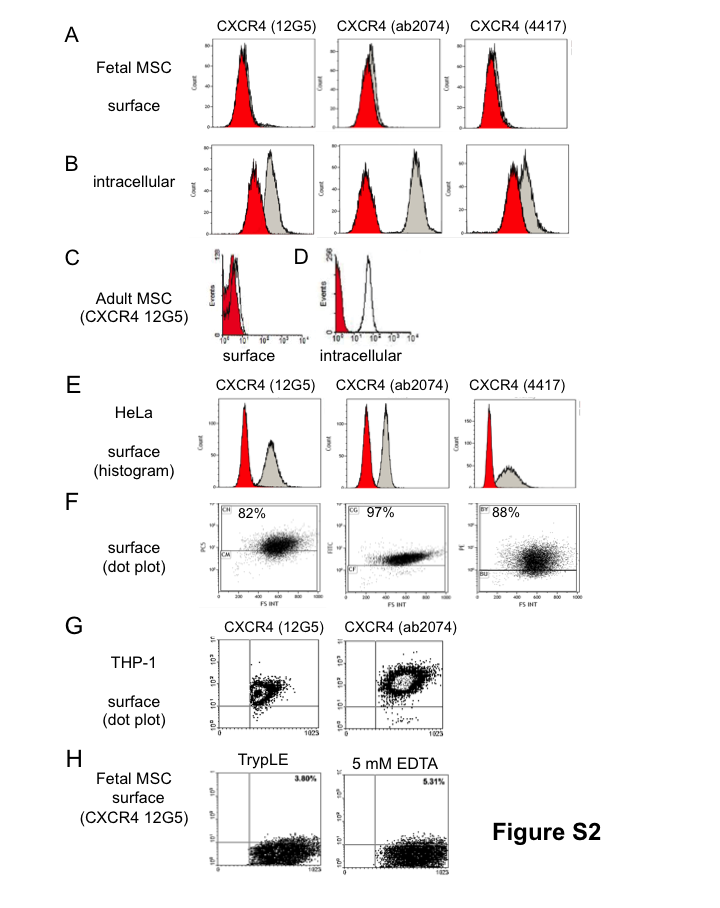

Supplement: Additional file 3: Figure S2 — Flow cytometry of CXCR4 expression by MSC control cell lines. Three different anti-CXCR4 antibody clones were used 12G5, ab2074 and 4417 as detailed in the methods, with fluorophore labelled secondary antibodies used where necessary. A) Fetal MSC CXCR4 surface expression (cell count vs fluorescence intensity) by flow cytometry shown as histograms plots (same data as for Figure 3). B) After permeabilization, the majority of cells show intracellular stores of CXCR4. Red histogram indicates isotype control. Human adult bone marrow MSC also show low expression of CXCR4 on the cell surface (C) but large intracellular store of this receptor (D). E and F) All the anti-CXCR4 antibodies are able to detect >80% cells with surface expression of CXCR4 on HeLa human cervical cancer cells. G) Both the anti-CXCR4 antibodies 12G5 and ab2074 are able to detect >80% cells with surface expression of CXCR4 on THP-1 human monocytic leukaemia cells. H) Fetal MSC were detached with TrypLE trypsin replacement or 5 mM EDTA and stained with CXCR4 (12G5) antibody to assess the effect of enzymatic dissociation on the number of cells staining positive for surface CXCR4. [file 1471-2121-15-15-S3.tiff]

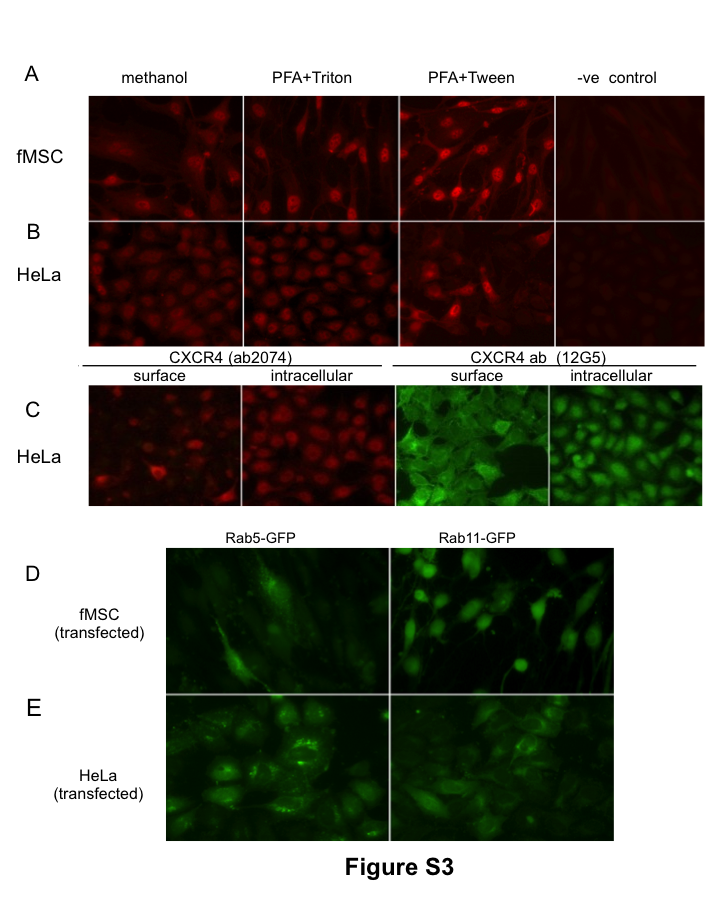

Supplement: Additional file 4: Figure S3 — Optimization of the fixation and permeabilisation conditions for the anti-CXCR4 (ab2074) antibody in fMSC or HeLa cells. (A and B) fMSC or HeLa cells were fixed and permeabilised as follows (from left to right): methanol, 4% paraformaldehyde (PFA) + 0.3% Triton and PFA + 0.1% Tween, and then stained with CXCR4 (ab2074) as detailed in the methods section. The negative control was PFA + Triton treated cells but the primary CXCR4 antibody omitted. C) HeLa cells with the anti CXCR4 antibody clone ab2074 or 12G5, where the cells were fixed in PFA to show surface staining or treated with PFA + Triton to show intracellular staining. D and E) Fetal MSC and HeLa cells were transiently transfected with Rab5-GFP or Rab11-GFP constructs to ensure the accuracy of the Rab antibody staining. Note that Rab5-GFP shows classic punctate endosomal localization in both fMSC and HeLa, whereas the Rab11 shows diffuse cytoplasmic localization in fMSC and classical endosomal punctae in the HeLa cells, similar to antibody staining in Figure 2. [file 1471-2121-15-15-S4.tiff]

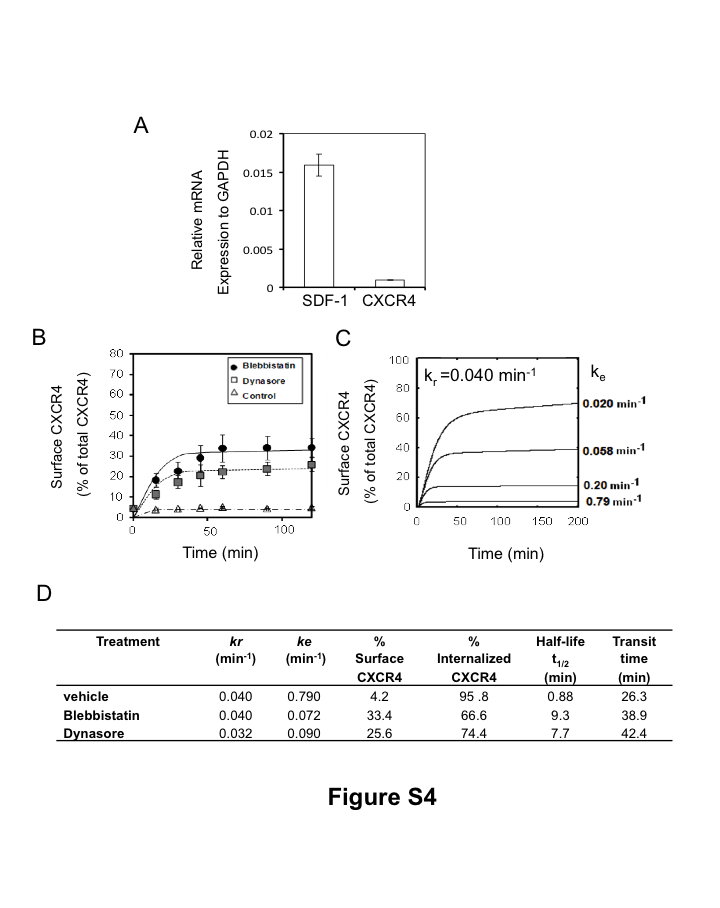

Supplement: Additional file 5: Figure S4 — Two compartment modelling of CXCR4 trafficking. (A) Real time PCR for SDF-1: Expression of SDF-1 in fMSC (n = 4) shows SDF-1 and CXCR4 transcripts relative to the housekeeping gene GAPDH. B) Kinetics of CXCR4 exocytosis in fMSC after treatment with endocytosis inhibitors: Cells were treated with 80 μM blebbistatin or 80 μM dynasore then fixed and stained with anti-CXCR4. Surface expression was determined by flow cytometry and data fitted to a two-compartment model of endocytosis (same data as Figure 1C). C) Fit of CXCR4 surface expression data for naïve (kr = 0.04 min−1, ke = 0.79 min−1), blebbistatin- (kr = 0.04 min−1, ke = 0.078 min−1, r2 = 0.89) and dynasore-treated cells (kr = 0.03 min−1, ke = 0.09 min−1, r2 = 0.91) to the two compartment model. Simulation was initiated with cytoplasm (endosomes) containing the entire cellular CXCR4. Response of surface expression to changes in the endocytosis rate (ke): kr was maintained at 0.04 min−1 while ke was varied from 0.79 min−1 to 0.02 min−1. D) Data that was used in the mathematical modelling of CXCR4 trafficking. [file 1471-2121-15-15-S5.tiff]

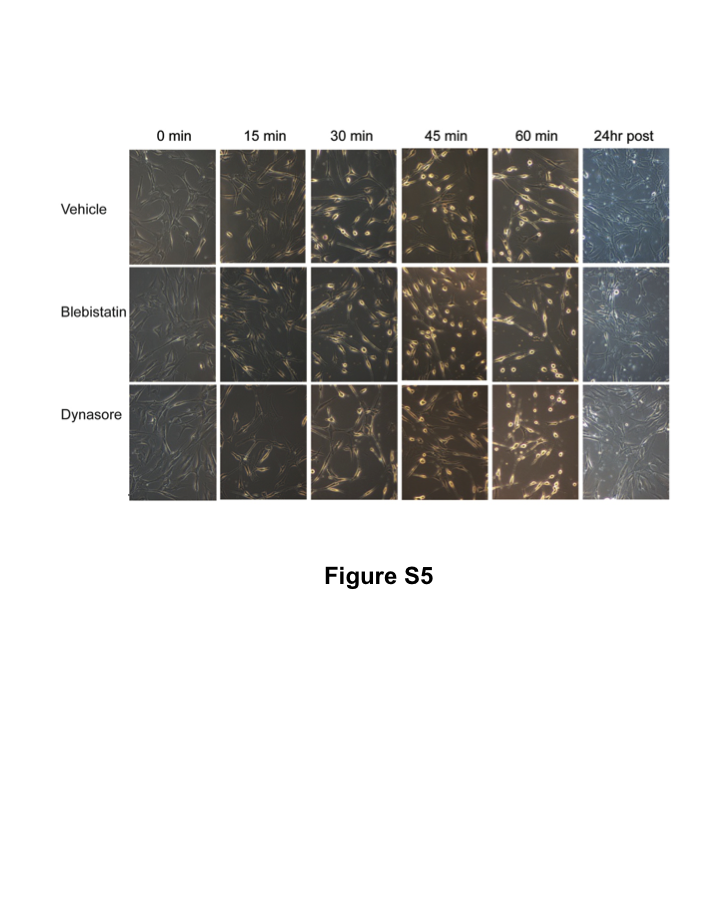

Supplement: Additional file 6: Figure S5 — Endocytosis inhibitors have a rapid but transient effect on fMSC morphology. Light microscopy images show morphology of fMSC rapidly changes from wide flattened fibroblast-like state (far left panels, 0 min) to cells that become rounded in the centre with long projections after vehicle (DMSO), blebbistatin and dynasore treatment. Cell morphology returns to normal by 24 hr post treatment and removal of the reagents (x10 magnification). [file 1471-2121-15-15-S6.tiff]

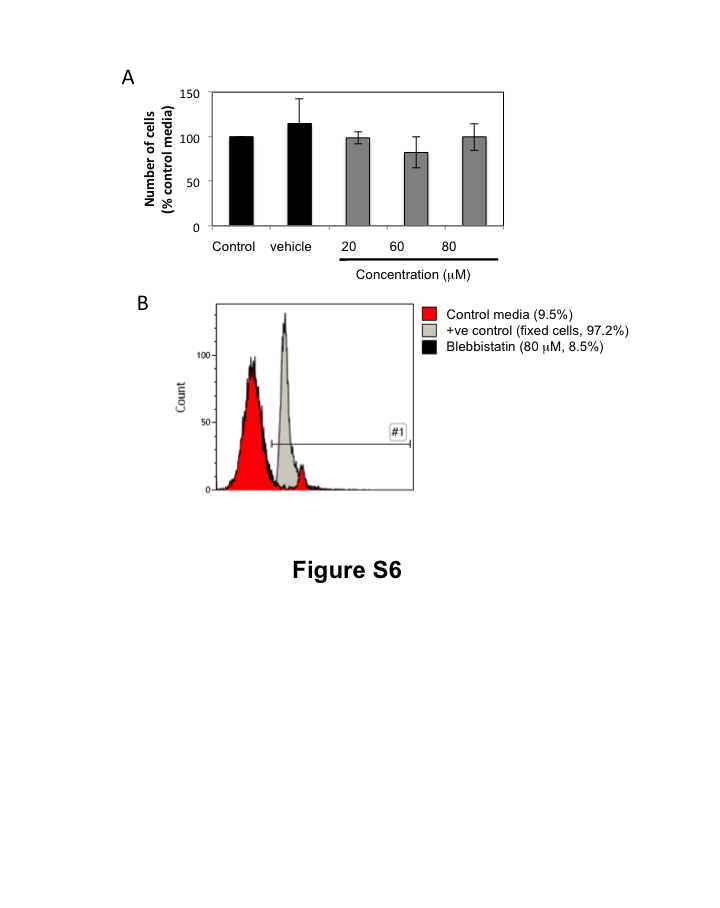

Supplement: Additional file 7: Figure S6 — Comparison of fMSC survival after inhibitor treatment. A) The Picogreen assay showed that there was no significant difference in the number of cells in control (serum free media), vehicle (0.45% DMSO) conditions or with increasing dose of blebbistatin for 1 hr. B) 7-AAD dye exclusion analysed by flow cytometry showed no significant difference between control media (vehicle) and blebbistatin treated fMSC after 1 hr incubation. [file 1471-2121-15-15-S7.tiff]
